# The German Dementia Registry (DEMREG): study protocol of a biomarker-based national registry for cognitive impairment and dementia

**DOI:** 10.1186/s42466-025-00433-9

**Published:** 2025-10-27

**Authors:** Kathrin Reetz, Ana Sofia Costa, Jennifer Michels, Milena Albrecht, Pia Moschko, Jennifer Pahl, Alexa Häger, Rainer Schuckelt, Rainer Röhrig, Jan Wienströer, Agnes Flöel, Emrah Düzel, Oezguer A. Onur, Timo Grimmer, Johannes Levin, Lutz Frölich, Frank Jessen, Jörg B. Schulz, The German Dementia Registry (DEMREG) Study Group, Dörte Polivka, Thomas Duning, Alexander Gutschalk, Timo Oberstein, Marlena Schnieder, Iris Trender-Gerhard, Christoph Laske, Volker Dahling, Oliver Peters, Dorothee Saur, Eike Spruth, Bastian Cheng, Fabian Fußer, Thorsten Bartsch, Wenzel Glanz, Lucrezia Hausner, Matthias Riemenschneider, Esther Höltje, Julian Hellmann-Regen

**Affiliations:** 1 Memory and Prevention Centre, Aachen, Germany; 2 Department of Neurology, RWTH Aachen University, Aachen, Germany; 3 JARA-BRAIN Institute Molecular Neuroscience and Neuroimaging, Forschungszentrum Jülich GmbH and RWTH Aachen University, Aachen, Germany; 4 Clinical Trial Center (CTC-A), RWTH Aachen University, Aachen, Germany; 5 Institute of Medical Informatics, RWTH Aachen University, Aachen, Germany; 6 Department of Neurology, University Medicine Greifswald, Greifswald, Germany; 7 German Center for Neurodegenerative Diseases (DZNE), Greifswald, Germany; 8 Institute of Cognitive Neurology and Dementia Research Otto-Von-Guericke University Magdeburg, Magdeburg, Germany; 9 German Center for Neurodegenerative Diseases (DZNE) Magdeburg, Magdeburg, Germany; 10 Department of Neurology, Faculty of Medicine and University Hospital Cologne, University of Cologne, Cologne, Germany; 11 Department of Psychiatry and Psychotherapy, School of Medicine and Health, TUM University Hospital, Technical University of Munich, Munich, Germany; 12 Department of Neurology, Ludwig-Maximilians-Universität München, Munich, Germany; 13 German Center for Neurodegenerative Diseases (DZNE), Munich, Germany; 14 Munich Cluster for Systems Neurology (SyNergy), Munich, Germany; 15 Department of Geriatric Psychiatry, Central Institute of Mental Health, Medical Faculty Mannheim, University of Heidelberg, Mannheim, Germany; 16 Department of Psychiatry, Medical Faculty, University of Cologne, Cologne, Germany; 17 German Center for Neurodegenerative Diseases (DZNE), Bonn-Cologne, Germany; 18 Department of Neurology, University Hospital Ulm, Ulm, Germany; 19 Department of Neurology, University Hospital Bremen-Ost, Bremen, Germany; 20 Department of Neurology and Policlinic, University Hospital Heidelberg, Heidelberg, Germany; 21 Psychiatric and Psychotherapeutic Clinic, University Hospital Erlangen, Erlangen, Germany; 22 Clinic for Geriatrics, University Medical Center Göttingen, Göttingen, Germany; 23 Department of Neurology, University Hospital Essen, Essen, Germany; 24 Psychiatry and Psychotherapy, University Hospital Tübingen, Tübingen, Germany; 25 Center for Mental Health, Brandenburg Medical School Theodor Fontane, Immanuel Hospital Rüdersdorf, Neuruppin, Germany; 26 Psychiatric and Psychotherapeutic Clinic, Hospital Frankfurt (Oder), Frankfurt (Oder), Germany; 27 Department of Neurology, University of Leipzig Medical Center, Leipzig, Germany; 28 Psychiatric and Psychotherapeutic Clinic, Charité University Medicine Berlin, Berlin, Germany; 29 Clinic and Polyclinic for Neurology, University Hospital Hamburg Eppendorf, Hamburg, Germany; 30 Clinic for Geriatric Psychiatry, Psychosomatics and Psychotherapy, Pfalzklinikum Klingenmünster, Klingenmünster, Germany; 31 Clinic for Neurology Campus Kiel, University Hospital Schleswig-Holstein, Kiel, Germany; 32 University Hospital Magdeburg, Magdeburg, Germany; 33 Central Institute for Mental Health, Clinic for Geriatric Psychiatry, Mannheim, Germany; 34 Clinic for Psychiatry and Psychotherapy, Homburg, Germany; 35 Clinic for Gerontopsychiatry, Memory Clinic, Asklepios Klinik Nord, Ochsenzoll, Germany; 36 Clinic for Psychiatry and Psychotherapy, Charité University Medicine Berlin, Berlin, Germany

**Keywords:** Dementia, Registry, Biomarkers, Neurodegeneration, Natural history, Treatment, Mild cognitive impairment

## Abstract

**Introduction:**

The German Dementia Registry (DEMREG) is a large-scale national prospective biomarker-based study for cognitive impairment and dementia, providing an integrated clinical research platform for research studies.

**Methods:**

The DEMREG study longitudinally collects demographic, clinical, genetic, biological, and imaging data, along with risk factors and treatment information from real-world settings. Comprehensive clinical assessments are conducted yearly. This extensive resource enables researchers to investigate current diagnostic and treatment practices and explore the complex relationships between risk factors and outcomes. The registry is now active across 22 sites in Germany, all members of the the German Memory Clinic Network (DNG), with more than 500 patients recruited to date, and is expected to include up to 1.000 patients annually.

**Perspective:**

The DEMREG study represents a large nationally harmonized cohort of detailed real-world clinical and biological data from patients with cognitive impairment and dementia, enabling insights into long-term dynamics and treatment responses. This infrastructure has the potential to foster collaborative research and roll out healthcare innovations across different settings in Germany. In this context, a substudy will soon be conducted to evaluate long-term safety and efficacy measures of the new monoclonal antibodies targeting amyloid plaques in a clinical setting.

**Trial registration:**

The protocol is registered at German Clinical Trials Register (DRKS00027547), Date of Registration: 01.04.2022.

## Introduction

### Rationale

The focus of treatment development for Alzheimer’s disease (AD) has shifted toward the earlier stages of the disease, based on the assumption that pathological changes begin decades before symptoms appear. This shift has been facilitated by the continuous development of diagnostic and research criteria, which help identify prodromal phases by incorporating fluid and imaging biomarkers, thereby achieving improved diagnostic specificity in early disease stages. Additionally, the importance of specific biomarkers has also grown, as they are now essential for determining whether a patient qualifies for anti-amyloid antibody treatments. Although such changes highlight the critical role of biomarkers in both clinical practice and research related to AD diagnosis and management, there was still no biomarker-based registry for cognitive impairment and dementia in Germany. The German Dementia Registry (DEMREG, Deutsches Demenzregister, https://www.ukaachen.de/DemReg) is an initiative aimed at filling this gap by systematically collecting, managing, and analyzing data related to cognitive impairment and dementia. The data collected will provide longitudinal insights into the natural history of affected patients, as well as the current routine diagnostic and treatment practices within the German healthcare system. Ultimately, this initiative aims to improve the quality of life for patients and caregivers, while advancing the state of dementia research globally.

### Objectives

The primary goal of the DEMREG is to establish a register for the collection of data and biomarkers related to amyloid-beta (Aβ) and tau pathology of cognitive impairment and early dementia in Germany.

The central task of the DEMREG is to systematically collect longitudinal data on demographics, clinical and neuropsychological characteristics, as well as imaging, blood, and cerebrospinal fluid markers in patients with subjective cognitive decline (SCD), mild cognitive impairment (MCI) or mild dementia to measure the natural course and therapeutic effects.

It is designed to investigate the natural course of cognitive disorders, while also capturing the effects of therapeutic interventions and identifying risk factors that may influence disease progression. By integrating longitudinal clinical, biomarker, and treatment data from clinical routine, the registry aims to uncover patterns that contribute to a better understanding of disease dynamics. Ultimately, the findings are intended to support the development of targeted strategies to improve patient care and optimize treatment pathways.

These activities simultaneously promote scientific exchange and sharing of gathered data, as well as interdisciplinary cooperation. Additionally, the DEMREG also entails administrative support for research projects and consultation on project applications. A key focus is also fostering the development of young scientists and disseminating research findings through publication.

Together, DEMREG is dedicated to accelerating the development of therapeutics in dementia by compiling uniform clinical data and biological samples and providing an academic research platform for further research studies, ultimately fostering clinical care and improving health outcomes.

### Study design

The study is a registered (DRKS00027547; 01.04.2022) open-ended prospective registry.

## Methods: participants and outcomes

### Study setting

**Fig. 1:**
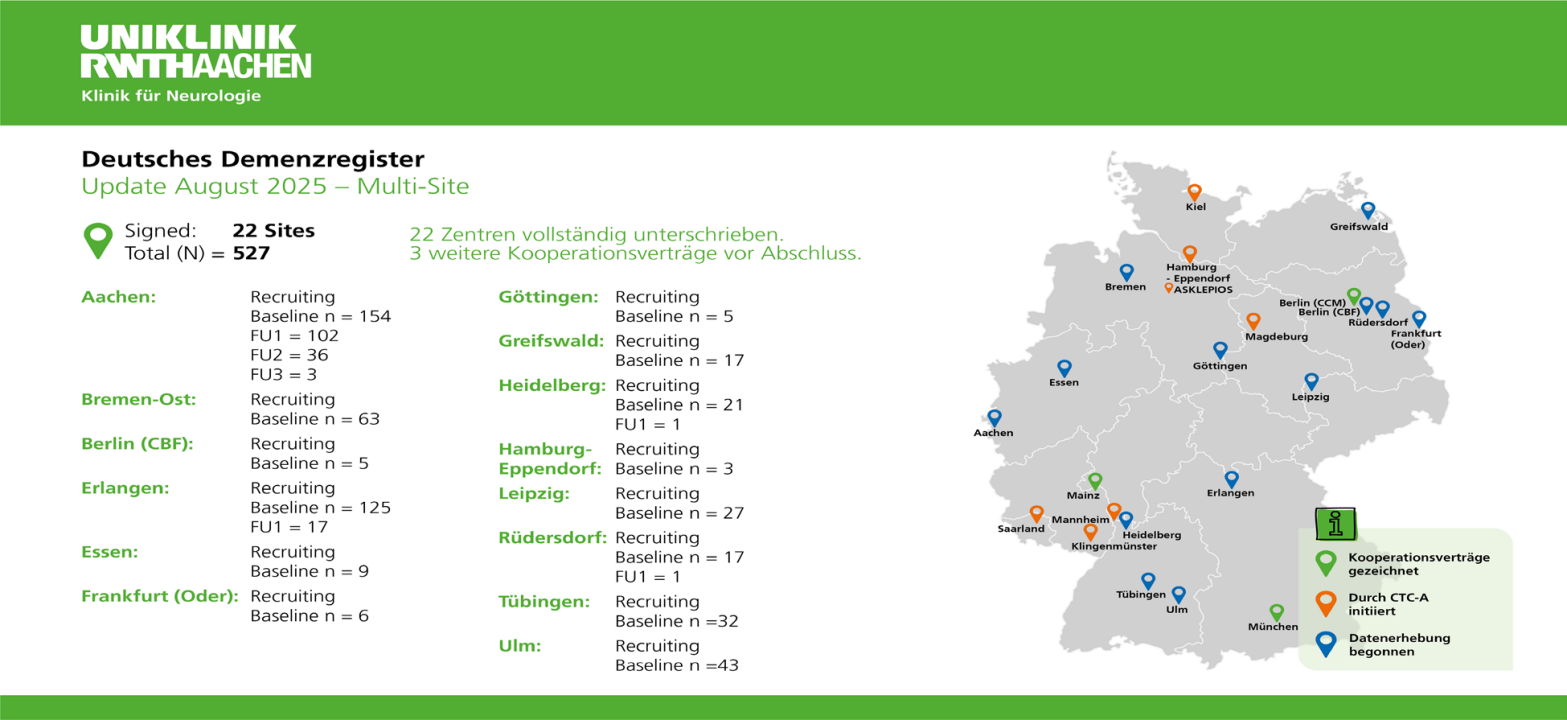
Overview of DEMREG study sites in Germany

All memory clinics within the German Network of Memory Clinics (Deutsches Netzwerk Gedächtnisambulanzen [DNG]; www.gedaechtnisambulanzen.de) are eligible to participate in this registry. Founded in Aachen in 2021, patient recruitment began in 2022. The multi-site expansion was launched in 2024, with currently 22 centers actively recruiting and collecting data, and five centers awaiting final ethical approvals and study initiation (Fig. 1; [status 27-August-2025], updates can be found on www.ukaachen.de/DemReg.

### Eligibility criteria

Patients are eligible as participants if: (a) they (or their legally authorized representative) are able to understand the purpose and risks of the registry and provide written informed consent and authorization to use protected health information in accordance with national and local privacy regulations; (b) they have received a diagnosis of subjective cognitive decline (SCD), mild cognitive impairment (MCI), or mild dementia, irrespective of etiology; (c) they have available results of neurodegeneration biomarkers (i.e., cerebrospinal fluid (CSF) Aβ_1-42_, Aβ_1-40_, Aβ_1-42_/Aβ_1-40_ ratio, total tau, and phosphorylated tau, and/or Aβ-PET or tau- PET imaging); and (d) they are 18 years of age or older. Study partners are eligible if: (a) they are 18 years of age or older, (b) they are study partners of a patient who has been included in the registry, (c) they have regular contact with the patient (according to the patient’s statement), and (d) they can understand the purpose and risks of participating in the study and provide written informed consent. Exclusion criteria for patients include the inability or unwillingness to provide written informed consent and the unavailability of biomarker results of Aβ and tau pathology. For study partners, the exclusion criterion is the inability or unwillingness to provide written informed consent.

### Informed consent

The registry was approved by the Ethics Committee of the Faculty of Medicine at RWTH Aachen University (EK 279/21) and for all participating sites. Written informed consent must be obtained from the patient or the patient’s legal representative, as applicable, in accordance with local practices and regulations. Written informed consent is also required for study partners. If the participant agrees to the storage of blood samples in the biobank (cBMB RWTH Aachen), a separate written informed consent must be obtained for this purpose.

### Outcomes and participant timeline

Outcomes have been selected based on their common use in memory clinics in Germany, as well as their clinical relevance and usage in clinical trials. In addition, harmonization procedures have been applied with the DESCRIBE registry of the DZNE (Deutsches Zentrum für Neurodegenerative Erkrankungen; https://www.dzne.de/forschung/studien/klinische-studien/describe/).

Visit schedules and outcome measures are illustrated in Table 1. Most of the data (e.g. demographics, medical history, medication, cognitive assessments) are collected from routine clinical records. Additional diagnostic parameters, such as biomarkers, imaging and laboratory results, will also be documented if they are available as part of the clinical routine. Existing neuropsychological data from routine clinical assessments that are no older than six months will also be included. If consented to, blood samples will be collected for biobank storage. Follow-up visits are conducted on an annual basis (± 3 months). As this is a registry, any variations from the proposed schedule will not be considered protocol deviations. To minimize any potential burden, participants can choose to take part in one of the following sets of assessments (see Table 1): (1) Core Visit (approximately 90 min), (2) Core + Extended Visit (approximately 105 min), or (3) Core + Extended + Optional Visit (approximately 120 min). Patients are encouraged to continue participating in the study for as long as possible. Visits are planned on an annual basis unless other sub-studies require other time intervals.

**Table 1:** DEMREG annual assessments and data elements

Data element/assessment	Core	Extended	Optional
Consent (Baseline only)	x		
Demographics, medical history, general & clinical information, risk-factors, treatments	x		
Diagnosis (syndromic and etiological)	x		
Mini-Mental-State-Examination (MMSE)	x		
Fluid Biomarkers^a^ (Aß1-42, Aß 1–40, Aß ratio, p-Tau, total Tau)	x		
CERAD-Plus Test Battery	x		
Montreal Cognitive Assessment (MoCA)	x		
Clinical Dementia Rating Scale (CDR)	x		
Geriatric Depression Scale (GDS-15)	x		
Alzheimer’s Disease Cooperative Activities-of-Daily-Living Scale for use in MCI (ADCS-ADL-MCI)		x	
Neuropsychiatric Inventory (NPI-12)		x	
European Quality of Life 5 Dimensions 5 Level Version (EQ5D-5L)		x	
ApoE Genotyping and Genetics			x^b^
Quality of Life in Alzheimer’s Disease (Qol-AD)			x
Zarit Burden Interview (ZBI)			x
Modified Perceived Deficits Questionnaire (MPDQ20)			x
Resource Utilization in dementia—Lite Version (RUD-Lite)			x
Short Form- 36Health Survey (SF-36)			x
MR –Imaging visual ratings			x
Degree of medial temporal atrophy (MTA)			
Degree of global cortical atrophy (GCA)			
Degree of parietal atrophy			
White matter hyperintensities (Fazekas)			
Presence of strategic infarcts			
Presence of microbleeds			
Presence of macrobleeds			
PET-Imaging			x
FDG-PET			
Aß-PET			
Tau-PET			
Biosample material			x

MR, magnetic resonance imaging; PET, positron emission tomography

^a^Cerebrospinal fluid (CSF) and/or blood-based

^b^obligate for Sub-study (s. Table 2)

### Sample size

This is an open-ended prospective registry with unrestricted recruitment and no fixed number of participants. It is expected that up to 1.000 patients will be included in the registry each year.

### Recruitment

Recruitment is ensured by the growing number of specialized memory clinics within the DNG.

### Data collection and management

Data (patient characteristics, medical history, risk factors, medication, neuropsychological tests) will be collected during the visits in specialized memory clinics. Additional diagnostic parameters (biomarkers, imaging, laboratory parameters) will be documented. Data are collected via a web-based eCRF. The electronic database is monitored remotely on a regular basis.

### Demographics

The following demographic information will be documented for each patient: year of birth, sex (male/female/non-binary), ethnicity, International Standard Classification of Education (ISCED) level, family history of dementia in first-degree relatives (coded as negative/positive/not informative), and marital status (unmarried/married/in a partnership/widowed/divorced).

### Clinical characteristics and parameters

Age in years at the time of symptom onset will be collected at baseline. For each patient, the following diagnostic information will be collected for each visit: level of syndromal impairment (SCD, MCI, mild/moderate/severe dementia) and etiological diagnosis (e.g., AD, vascular dementia, frontotemporal dementia) according to national diagnostic guidelines—S3 Guidelines for Dementia of German Societies of Neurology and Psychiatry [1]. Other medical diagnoses are entered as ICD-10 codes and free text. In each visit, systolic and diastolic arterial blood pressure, height in meters and weight in kilograms will be documented. Tobacco smoking status (no/yes/ex-smoker) and pack-years will also be documented. Physical activity in minutes per week will be coded according to predefined categories (no activity/ < 30 min/30–60 min/60–90 min/ > 90 min).

### Treatment

At all visits, current medication, including AD-specific medication as well as non-pharmacological interventions (e.g. physiotherapy/occupational therapy/speech and language therapy/digital health applications), will be documented.

### Cognitive, functional and neuropsychiatric outcomes

Total uncorrected results will be documented for the Mini-Mental State Examination (MMSE) [2] and Montreal Cognitive Assessment (MoCA) [3]. For the Clinical Dementia Rating scale (CDR) [4, 5], ratings for each of the six domain boxes will be collected (memory, orientation, judgment and problem solving, community affairs, home and hobbies and personal care). Other measures include the CERAD-Plus (Consortium to Establish a Registry for Alzheimer’s Disease including Trail Making Test A + B, phonemic fluency) [6] and the Alzheimer’s Disease Cooperative Study Activities of Daily Living scale for MCI-patients (ADCS-ADL-MCI) [7]. Neuropsychiatric outcomes include the total score of the 15-item version of the Geriatric Depression Scale (GDS) [8] and the Neuropsychiatric Inventory (NPI-12). Patient-reported outcomes (PROMs) also include the Perceived Deficits Questionnaire (PDQ), the Quality of Life in Alzheimer’s Disease (QOL-AD), and the 36-Item Short Form Health Survey (SF-36). Proxy-reported outcomes include the Resource Utilization in Dementia lite (RUD-lite) [9] and the Zarit Burden Interview (ZBI) [10].

### Brain imaging outcomes

For available routine brain MRI-scans, the following rating scales are used to semi-quantify atrophy and white matter changes: bilateral mesial temporal atrophy (MTA) score [11], global cortical atrophy score (GCA) [12], parietal atrophy score (Koedam score) [13], age-related white matter changes score (ARWMC rating for periventricular white matter lesions and deep white matter lesions) [14]. Presence of strategic infarcts, macrohemorrhages and/or microhemorrhages (including number for each location—deep, infratentorial and cortical), as well as of amyloid-related imaging abnormalities with cerebral edema (ARIA-E) and/or with cerebral hemorrhages (ARIA-H) are also documented. When available, results from functional imaging examinations, namely [18F] FDG-PET, DaTSCAN, Aβ -PET, and/or tau-PET are also documented.

### Fluid markers

The following CSF-parameters are collected Aβ1–42, Aβ1–40, Aβ 1–42/1–40 ratio, total tau (t-tau) protein, phosphorylated tau protein181 (p-tau). For each study site the respective reference values are collected and the platforms on which the CSF-measures are obtained can be entered into the database. The analyses are conducted using commercially available assays for the determination of neurodegenerative markers, which have been validated in clinical populations, and have demonstrated good diagnostic accuracy for AD. The pathological reference values in this case include Aβ1–42 < 450 pg/ml; Aβ ratio < 0.5; t-tau > 450 pg/ml; and p-tau > 61 pg/ml [15, 16]. If available, Aβ and tau blood biomarkers and respective references are also collected. Given the flexible EDC-system, potential new parameters can be added after protocol amendment.

## APOE-genotyping and genetics

Results of APOE-genotyping and genetic testing—such as APP, PSEN1, PSEN2, MAPT, GRN, C9orf72—will be recorded, if available.

### Biosamples

The RWTH centralized Biomaterial Bank (cBMB) of the RWTH Aachen University will be used as the biobank for this registry. Samples collected at other study sites will be sent in a timely manner after collection. The Steering Committee of the registry, which has a simple majority quorum, decides on access to the biosamples. The collection of biosamples is documented in the participants’ electronic case report form (eCRF). The following biosamples will be stored in the biobank: whole blood aliquots, plasma aliquots, and serum aliquots. Standard Operating Procedure (SOP) manuals are available to ensure biosample processing, including storage at − 80 °C, and shipping procedures.

### Sub-studies

One planned sub-study refers to the assessment of patients undergoing recently approved treatments, such as the upcoming anti-Aβ antibody therapies (e.g. Lecanemab/Donanemab). Procedures for this type of substudy involve a comprehensive evaluation process at pre-specified treatment visits, that include monitoring adverse events, brain imaging, and various clinical outcomes and PROMs (see Table 2). Next to details about the medication administration (e.g. dosage), potential adverse effects such as headache, nausea, dizziness, and infusion-related reactions will be documented. Data on the brain MRI will focus on the presence and severity assessment of ARIA-E (fluid hyperintensity) and ARIA-H (microhemorrhages and superficial siderosis), graded as none, mild, moderate, or severe. Brain imaging is performed at the start of treatment and pre-specified follow-up intervals, according to treatment approval guidelines. Data on possible additional brain imaging with the aim of managing adverse effects will be assessed using the same procedures. If available, additional imaging assessments include Aβ-PET scans, which quantify Aβ levels using centiloids, and Tau-PET scans that utilize standardized uptake value ratios (SUVRs) to evaluate tau pathology. Moreover, clinical assessments to assess clinical progression are done every six months, including the MMSE to monitor cognitive function and the EQ-5D-5L questionnaire to evaluate health-related quality of life. This structured approach ensures thorough monitoring of treatment effects, adverse events, and disease progression over time.

**Table 2:** Sub-study novel treatments

A) Special treatment assessment	Treatment visit
Treatment	
ApoE Genotyping	Baseline only
Adverse Events	
MR Imaging including radiological severity	
Baseline	
Follow-up	
ARIA-E Flair hyperintensity (none, mild, moderate, severe)	
ARIA-H microhemorrhage (none, mild, moderate, severe)	
ARIA-H superficial siderosis (none, mild, moderate, severe)	
Amyloid-PET^#^ using Amyloid levels in Centiloids	
Tau-PET^#^ using standardized uptake value ratios (SUVRs)	
EQ 5D-5L questionnaire	Every 6 months
Mini-Mental State Examination (MMSE)	Every 6 months

MR, magnetic resonance imaging; PET, positron emission tomography; # if available

### Plans to promote participant retention and complete follow-up

In general, participation in the study is associated with routine clinical visits to reduce the burden on participants. Patients may be withdrawn from the registry for any of the following reasons: (a) the patient withdraws consent; (b) the physician withdraws the patient from the registry for medical reasons; or (c) the patient is placed under legal guardianship due to the increased severity of the illness, and the guardian does not agree to further participation. The reason for the patient’s withdrawal from the study will be dated and recorded in the patient’s eCRF.

## Data management and confidentiality

Data are entered and managed via an eCRF using LibreClinica, an open-source web-based electronic data capture (EDC) tool. Data entry is double-checked by each study site, with automatic range checks. Data are stored on an in-house server at the RWTH Aachen University Hospital. Protections against unauthorized access comply with the Federal Office for Information Security (www.bsi.bund.de) standards. Access to personal data is limited to authorized personnel for quality assurance, who are bound to confidentiality. After data quality checks, personal identifiers are fully anonymized, with only limited linking information kept securely. Data protection follows the German Federal Data Protection Act. Participants can review and correct their data, request information, or lodge complaints with authorities. If a participant withdraws, their data is fully anonymized. All study documents are stored securely for up to ten years after the closure of the registry. The Department of Neurology will provide access to the data according to the FAIR data principles.

### Statistical methods

#### Methods of analyses

The registry’s primary goal is to evaluate current diagnostic and treatment practices in terms of their effectiveness and influence on disease progression, and to monitor the natural course of disease progression in a cohort of patients with cognitive impairment and dementia in Germany. The statistical analysis plan (SAP) includes an initial analysis of baseline characteristics of a German memory clinics’ cohort, which will be primarily descriptive, and with larger datasets enabling advanced statistical methods—such as linear mixed-effects models to assess progression rates and the validity of outcomes (e.g. responsiveness) on longitudinal assessments. Continuous variables will be reported using measures like mean, standard deviation, range, and quantiles, with categorical data presented as counts and percentages of valid responses.

### Oversight and monitoring

#### Composition of the coordinating center and steering committee

A Steering Committee was established to provide scientific and medical direction to the registry and to oversee the administrative progress of the study. The Steering Committee will meet at appropriate intervals to monitor patient recruitment and compliance with the protocol. The Steering Committee will decide whether the study should be stopped or modified. The Steering Committee will also decide on requests for the use of the data. Data may be exported in pseudonymized form if the request is for scientific purposes. If there is a commercial request, the data will only be released in anonymized or aggregated form. The Steering Committee is formed by a vote of the study sites and comprises nine members. The members are elected by the assembly of cooperation partners.

### Monitoring

The data are monitored remotely on a regular basis by the data monitoring team. Participant-level data are subject to remote quality checks that control cross-sectionally and longitudinally for consistency, completeness, and plausibility. In addition, site-level monitoring of compliance, performance, and data quality is performed. Data quality audits will identify missing, incorrect, or inconsistent entries, with trained personnel reviewing queries to detect error trends and determine the need for additional site training. Data completeness will be monitored biannually, and descriptive statistics will summarize patient demographics and disease characteristics. All sites are routinely monitored to review source data and ensure compliance with the study protocol, Good Clinical Practice (GCP), and other applicable regulations. Tailored feedback and support are provided to improve underperforming sites and corrective action plans are implemented when appropriate. To complement and support regular remote monitoring, a process-oriented monitoring will be implemented.

### Plans for communicating important protocol amendments to relevant parties

As required by local law, any amendments to the protocol that affect patient safety, the scope of the study, or the scientific quality of the study will be submitted for approval by the ethics committees prior to their implementation.

### Dissemination plans

The dissemination plan will share registry findings via peer-reviewed publications, conferences, and stakeholder engagement to ensure broad visibility. Results will be communicated to clinicians, policymakers, patients, and the public through online platforms, webinars, and summaries. Data sharing will be conducted responsibly to support further research and collaboration, maximizing the impact on clinical practice and health policy.

### Perspective

DEMREG is a nationwide registry for cognitive impairment and dementia, offering a large, real-world cohort with detailed clinical and biological data. Built on a flexible EDC system, it supports multiple studies, long-term monitoring, and easy participant entry. It enables recruitment for trials, fosters biomarker and outcome research, and provides infrastructure and expertise for industry and academic partners to assess feasibility, identify sites, and plan studies.

### Study status

Protocol version number 5.0 from 18.11.2024. Start of recruitment: 16.05.2022. Multi-site expansion in 2024.

### Contacts

The study is being conducted in close collaboration with the Clinical Trial Center Aachen (CTC-A) and with the support of the Institute for Medical Informatics (IMI). It is financially supported by private sponsors, industry partners Biogen GmbH, Eisai GmbH, and Lilly Deutschland GmbH.

## Data Availability

The data will be deposited on a protected server of the RWTH Aachen University Hospital. Access is strictly regulated, even for study personnel. Owing to the difficulty of de-identification (routine care, qualitative data, etc.), individual participant data will not be shared publicly. Upon reasonable request, which includes a methodologically sound proposal for the usage of data and approval by the responsible review committee, data may be shared.

## Abbreviations

AD: Alzheimer’s disease
ADAS-Cog: Alzheimer’s disease assessment scale-cognitive subscale
ADCS-ADL-MCI: Alzheimer’s disease cooperative study activities of daily living scale for MCI patients
BSI: Bundesamt für Sicherheit in der Informationstechnik
cBMB: Centralized biomaterial bank
CERAD-PLUS: Consortium to establish a registry for Alzheimer’s disease- Plus test battery
CDR: Clinical dementia rating scale
CSF: Cerebrospinal fluid
CTC-A: Clinical Trial Center Aachen
DEMREG: German Dementia Registry
eCRF: Electronic case report form
EDC: Electronic data capture
GCA: Global cortical atrophy
GCP: Good clinical practice
GDS: Geriatric depression scale
ICF: Informed consent form
MCI: Mild cognitive impairment
MMSE: Mini-Mental State Examination
MoCA: Montreal cCgnitive Assessment
MRI: Magnetic resonance imaging
MTA: Mesial temporal atrophy
NPI: Neuropsychiatric inventory
PET: Position emission tomography
PDQ: Perceived deficits questionnaire
PROMs: Patient-reported outcome measures
QOL-AD: Quality of life in Alzheimer’s Disease
RUD lite: Resource utilization in dementia lite
SAP: Statistical analysis plan
ZBI: Zarit burden interview
